# Gut microbiota in early pregnancy among women with Hyperglycaemia vs. Normal blood glucose

**DOI:** 10.1186/s12884-020-02961-5

**Published:** 2020-05-11

**Authors:** Beibei Gao, Mengdan Zhong, Qiong Shen, Ying Wu, Mengdie Cao, Songwen Ju, Lei Chen

**Affiliations:** 1 Department of Endocrinology, The Affiliated Suzhou Hospital of Nanjing Medical University, Suzhou Municipal Hospital, Suzhou, 215000 China; 2 Central Laboratory, The Affiliated Suzhou Hospital of Nanjing Medical University, Suzhou Municipal Hospital, Suzhou, 215000 China

**Keywords:** Hyperglycaemia in early pregnancy, Gut microbiota, 16S rDNA, Next generation sequencing

## Abstract

**Background:**

Recent studies suggest that there is a link between the gut microbiota and glucose metabolism. This study aimed to compare the gut microbiota during early pregnancy of women with hyperglycymia to those with normal blood glucose.

**Methods:**

Gut microbial composition was analysed in 22 women with hyperglycaemia and 28 age-matched healthy controls during their first prenatal visits (< 20 weeks) using high throughput sequencing of the V3-V4 region of the 16S ribosomal RNA gene. Hyperglycemia was diagnosed based on the criteria recommended by the International Association of Diabetes and Pregnancy Study Groups in 2010.

**Results:**

Women with hyperglycemia in pregnancy (HIP) had significantly lower microbial richness and diversity compared with healthy pregnant women. The proportions of the *Firmicutes* and *Bacteroidetes* phyla and the ratio of *Firmicutes*:*Bacteroidetes* were not different between the two groups. We observed that individuals with HIP had an increased abundance of *Nocardiaceae*, *Fusobacteriaceae*, etc., whereas healthy controls had significantly higher levels of *Christensenellaceae*, *Clostridiales_vadinBB60_group*, *Coriobacteriaceae*, etc. Similarly, levels of the members of the *Ruminococcaceae* family*,* including *Ruminococcaceae_UCG-014*, *Ruminococcaceae_UCG-003*, and *Ruminococcaceae_UCG-002,* were significantly reduced in the HIP group and were negatively correlated with HbA1c. HbA1c levels were positively correlated with *Bacteroidaceae* and *Enterobacteriaceae* and negatively correlated with *Christensenellaceae*, etc. CRP was positively correlated with the *Bacteroidaceae* and *Fusobacteriaceae* families and the *Fusobacterium* genus.

**Conclusions:**

Our study revealed that individuals with HIP have gut microbial dysbiosis and that certain bacterial groups are associated with glucose metabolism during pregnancy. Further study is needed to provide new ideas to control glucose by modifying the gut microbiota.

## Background

Hyperglycaemia in pregnancy (HIP) is a very common medical disorder during pregnancy that consists of two categories: pregestational diabetes (PGDM), antedating pregnancy, and gestational diabetes mellitus (GDM), initially diagnosed during gestation [1]. The increasing prevalence of HIP and its related adverse pregnancy outcomes are a global health concern [2–4]. Dysbiosis in the human gut may be a vital risk factor for abnormal glucose metabolism and was first discovered by Backhed F and his colleagues in 2004 who found that conventionalisation of germ-free mice with microbiota from conventionally raised animals produced a marked increase in body fat content and insulin resistance despite decreased food intake [5]. Since then, the association between the gut microbiota and glucose metabolism has been a popular research topic.

Data from human and animal studies suggest that individuals with obesity and insulin resistance have a lower bacterial diversity [6, 7]. A lower prevalence of the *Bacteroidetes* phylum and a higher prevalence of the *Firmicutes* phylum have been shown to be associated with obesity [8, 9]. Obesity was linked to changes in the *Bacteroidetes*/*Firmicutes* ratio, which has been associated with an increased capacity to harvest energy from the diet [9]. *Prevotella copri* and *Bacteroides vulgatus* are the main species associated with the biosynthesis of branched-chain amino acids, insulin resistance, and glucose intolerance [10]. Furthermore, the levels of butyrate-producing bacteria were reduced in diabetic individuals [10], and butyrate supplementation in obese, prediabetic mice significantly improved insulin resistance, hyperinsulinaemia, and hyperglycaemia [11].

While the majority of previous studies have been concerned with the associations between the gut microbiota and obesity or type 2 diabetes (T2DM) [8–10, 12, 13], some recent studies have explored changes in the microbiota during pregnancy. Decreased insulin sensitivity and enhanced nutrient absorption are beneficial for normal pregnancy, by supporting foetal growth and meeting the energetic demands of lactation [14]. Hormones, immunity and metabolism change substantially in pregnancy. However, human microbiota studies have also demonstrated that the gut microbiota changes dramatically during pregnancy [15]. From the first to third trimesters, *Bifidobacteria*, *Proteobacteria*, and lactic-acid-producing bacteria increase and butyrate-producing bacteria decrease [15].

What is the relationship between the gut microbiota and glucose metabolism disorders in the context of pregnancy? A study based on whole-metagenome shotgun sequencing discovered that the composition of the gut microbiota was different between controls and women with GDM at 21–29 weeks. GDM patients showed an increase in *Parabacteroides distasonis* and *Klebsiella variicola,* whereas controls showed an enrichment of *Methanobrevibacter smithii*, *Alistipes spp.*, *Bifidobacterium spp.*, and *Eubacterium spp.* [16]. Another recent study by Fangqing Zhao also demonstrated that GDM patients had lower microbial richness and diversity than controls within 1–2 days before delivery [17]. To date, there is no information available on the gut microbiota in relation to glucose metabolism disorder in early pregnancy.

In this study, we used 16S rRNA gene amplicon sequencing to analyse the gut microbiota in women with HIP and healthy controls during early pregnancy. We also explored the connections between gut microbiota composition and glucose metabolism-related indicators. The objective was to further understand the associations between the gut microbiota and HIP in early pregnancy.

## Methods

### Study population

In this study, 50 pregnant women in early pregnancy (< 20 weeks gestation) were recruited during their first prenatal visits at the Maternity and Child Health Center of Suzhou Municipal Hospital between 1 Nov 2015 and 31 Oct 2017. All participants were Han Chinese. Twenty-two pregnant women had different degrees of glucose metabolism disorders were signed into the HIP group. Among HIP group, 8 had type 2 diabetes mellitus before pregnancy who were only through diet/lifestyle to control glucose. Based on the diagnostic criteria recommended by the International Association of Diabetes and Pregnancy Study Groups (IADPSG) in 2010 [18], 5 overt diabetes and 9 GDM were diagnosed by hemoglobin A1c (HbA1c) or fasting glucose (FBG) and confirmed by an oral glucose tolerance test (OGTT). Twenty-eight pregnant women with fasting plasma glucose lower than 5.1 mmol/L were assigned into control group. Additionally, they had a normal glucose tolerance during 24–26 weeks’ gestation to comfirm they were healthy Patients were excluded if they met one of the following criteria: 1) history of antibiotic therapy within the last 3 months; 2) chronic diseases requiring medication; and 3) history of smoking. Written informed consent was obtained from all subjects before their participation. Ethics approval was obtained from the hospital.

### Characteristic data collection

The anthropometric data (pre-pregnancy weight (pre-weight), height, pre-body mass index (pre-BMI), waist circumference (waist) and hip circumference (hip)), lifestyle factors and history of medication and treatments were obtained from clinical medical records. Pre-BMI was calculated as the subject’s pre-weight (kg) divided by their height squared (m^2^). Waist and hip were measured in an erect position at the narrowest point between the iliac crest and the lower costal margin and at the level of the pubic symphysis, respectively. Systolic and diastolic blood pressure was measured twice (YuTu Model XJ11D, YuTu Company, Shanghai, China) with the participant in a sitting position, and the mean value was used for further analysis. The subjects were given instructions by a physician to how to be fasting prior to the collection of samples. After 8 h of fasting, venous blood samples were collected between 07:00 and 09:00 for the assessment of metabolic biomarkers (FBG, TC, TG, LDL-C, HDL-C, etc) using a fully automatic biochemical analyser (Hitachi 7000, Tokyo, Japan). HbA1c level was detected by using high-performance liquid chromatography (HLC-723G8, Tosoh Bioscience, Japan) Insulin content was measured using a Human Insulin ELISA Kit (Sigma-Aldrich, St. Louis, MO, USA).

### Sample collection, DNA extraction and 16S rRNA PCR

Fresh stool samples were collected from pregnant women at home using sterile faecal collection tubes, and samples were immediately transferred to the lab within 6 h and then stored at − 80 °C under a uniform protocol before DNA extraction. Total microbial genomic DNA from 250 mg stool was extracted using the Qiagen QIAamp DNA Stool Mini Kit (Qiagen, Shanghai, China) following the manufacturer’s protocols. A NanoDrop 2000 UV-vis spectrophotometer (Thermo Scientific, Wilmington, USA) and 1% agarose gel were employed to evaluate the purification and quality of the DNA samples. The V3-V4 hypervariable regions of the bacterial 16S rRNA gene were amplified using 338F and 806R primers on a thermocycler PCR system (GeneAmp 9700, ABI, USA). PCR reactions (20 μL: 4 μL of 5 × FastPfu Buffer, 2 μL of 2.5 mM dNTPs, 1.6 μL of 5 μM primers, 0.4 μL of FastPfu Polymerase and 10 ng of template DNA) were performed according to the following cycling programme: denaturation at 95 °C for 3 min; followed by 27 cycles of 95 °C for 30 s, 55 °C for 30 s, and 72 °C 45 s; and 72 °C for 10 min. Each sample was analysed in triplicate. PCR products with clear electrophoresis strips and an expected size were extracted from 2% agarose gel, purified using the AxyPrep DNA Gel Extraction Kit (Axygen Biosciences, Union City, CA, USA) and quantified using QuantiFluor™-ST (Promega, Madison, WI, USA).

### Illumina MiSeq sequencing and data processing

After purification and equivalent mixing of the PCR products, paired-end (2 × 300 bp) Illumina MiSeq (Illumina, San Diego, USA) sequencing was carried out at Nanjing Decode Genomics BioTech Co., Ltd. (Nanjing, China) according to the standard protocols. We trimmed and filtered the reads (FastQ files) by using Trimmomatic [19] and merged the paired reads with FLASH [20]. Operational taxonomic units (OTUs) were clustered using USEARCH [20, 21](version 7.1 http://drive5.com/uparse/) at the 97% similarity level. UCHIME was performed to filter out the chimeric sequences [22]. Taxonomic classification of OTUs was performed using the RDP Classifier algorithm [23] (http://rdp.cme.msu.edu/) by aligning representative sequences to the Silva (SSU123) 16S rRNA database [24].

### Statistical analysis

Continuous data are expressed as the median (interquartile range). Comparisons between groups were performed using Mann-Whitney *U* tests or Fisher’s exact test with adjusted age. The alpha diversity (including the Chao1 index and Shannon diversity index) of bacteria within individuals was used to evaluate the number of different taxa, species richness and evenness of bacterial taxa. Microbial community composition differences (beta diversity) between groups were analysed using permutational multivariate analysis of variance (PMANOVA) of unweighted or weighted UniFrac distances (Bray-Curtis distance method, 999 permutations), and the results are illustrated by principal coordinates analysis (PCoA). Canonical correspondence analysis (CCA) was used to assess the impact of each of the factors listed on microbial abundance. Differences in microbial abundance (at the phylum, family, genus and species levels) were analysed using the Mann-Whitney *U* test. *P*-values were adjusted for false discovery rate (FDR) using the Benjamini and Hochberg method. *P* < 0.05 was considered statistically significant. Bacterial community diversity and composition were illustrated using Boxplot graphs and cluster and heatmap diagrams in R (ggplot2 package). Statistical analyses were performed using Graph Pad Prism 6 software (GraphPad, San Diego, USA) or SPSS 19 software (IBM, New York, USA).

## Results

### Clinical characteristics and biochemical variables

The clinical characteristics of the 50 pregnant women at < 20 weeks gestation are presented in Table 1. Twenty-eight controls and 22 women with HIP were matched for maternal age. Fasting serum levels of seven biochemical variables (fasting blood glucose (GLU), triglycerides (TGs), high-density lipoprotein (HDL), low-density lipoprotein (LDL), HbA1c and C-reactive protein (CRP)) were measured in all participants. There were significant differences in GLU, TGs, HDL, LDL, HbA1c and CRP between the two groups, while TC did not show significant differences (Table 1), with subjects in the HIP group having a more disturbed metabolic profile.

**Table 1:** Characteristics of the study population

	HIP (*n* = 22)	Control (*n* = 28)	*P*-value
Age (years)	29 (27.00–33.5)	28 (25–30)	0.082
Pre-BMI (kg/m^2^)	24.28 (22.33–28.38)	20.49 (19.14–22.48)	0.000
Pre-weight (kg)	62.75 (55–73.5)	52.5 (49.00–58.5)	0.001
Waist (cm)	92.5 (86–106.5)	83.5 (76.0–86.5)	0.000
Hip (cm)	101.5(93.5–105.25)	92.5 (88.0–96.75)	0.002
SBP (mmHg)	134 (117.75–142.75)	119 (106.25–126.75)	0.002
DBP (mmHg)	83 (72–92)	69.5 (61.0–80.5)	0.004
FBG (mmol/L)	5.64 (5.2–7.9)	4.36 (4.05–4.56)	0.000
TC (mmol/L)	4.17 (3.97–4.68)	4.45 (3.83–4.85)	0.83
TGs (mmol/L)	1.77 (1.45–3.23)	1.18 (1.03–1.61)	0.000
HDL (mmol/L)	1.25 (1.09–1.53)	1.86 (1.61–2.04)	0.000
LDL (mmol/L)	2.71 (2.27–3.12)	2.32 (1.89–2.67)	0.047
HbA1c (mmol/L)	6.55 (6.1–7.88)	5.1 (4.93–5.3)	0.000
CRP (mmol/L)	4.75 (2.68–9.43)	2.09 (0.95–3.49)	0.002
FINS (mIU/L)	9.15 (6.7–16.55)	–	–

Values are represented as medians (interquartile range). Differences were analysed using Mann-Whitney U tests. *BMI* Body mass index; *SBP* Systolic blood pressure; *DBP* Diastolic blood pressure; *TC*, total cholesterol; *TGs*, triglycerides; *HDL*, high-density lipoprotein; *LDL* Low-density lipoprotein; *GLU* Fasting blood glucose; *FINS* Fasting insulin

### Bacterial community structure (beta diversity) in the two study groups

**Fig. 1:**
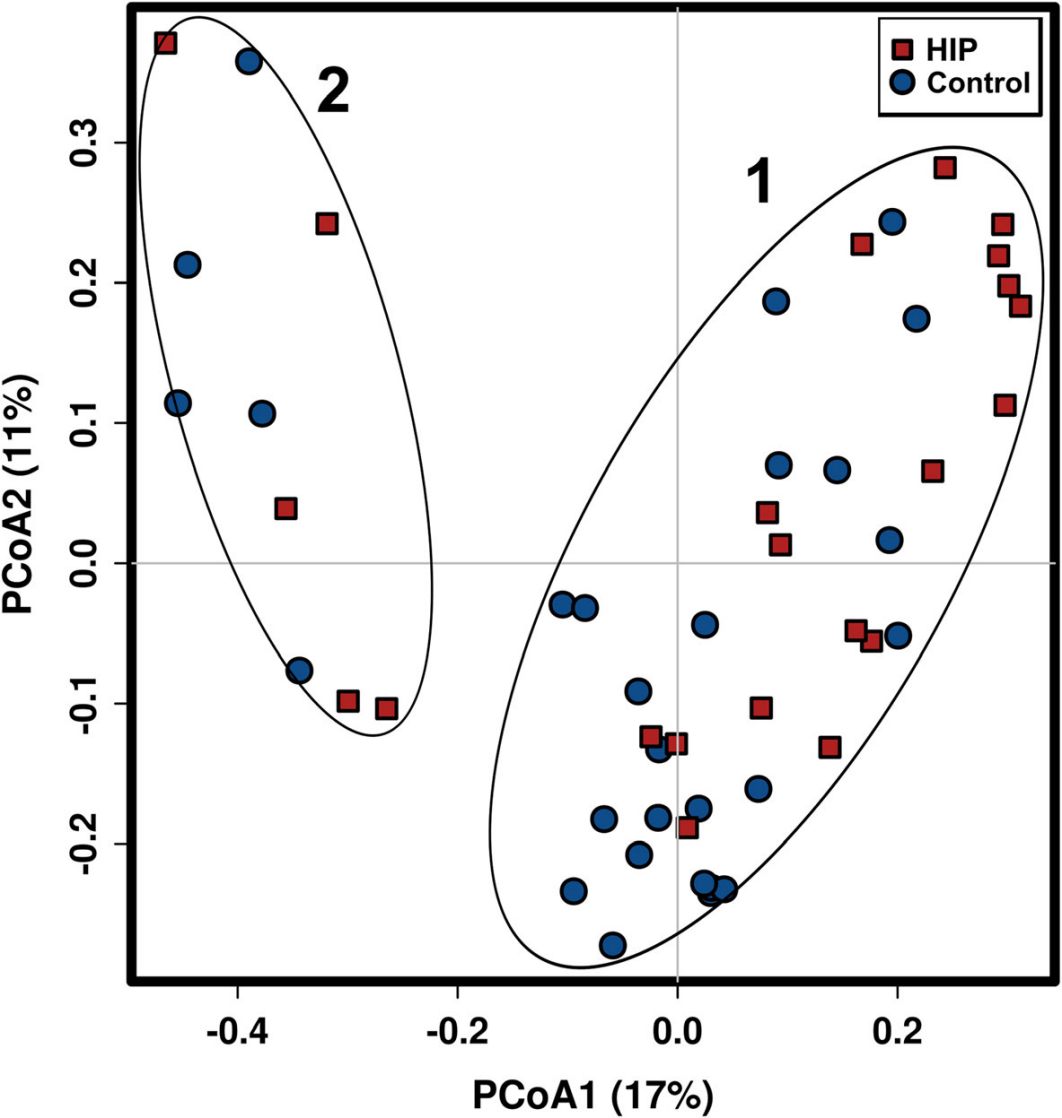
PCoA of the bacterial community composition based on Bray Curtis analysis. Dots represent individuals (women with HIP = red, controls = blue). Cluster 1 and Cluster 2 are represented as *Bacteroides* and *Prevotella* enterotypes, respectively

A total of 912 OTUs (with 97% similarity) were clustered in this study. The PMANOVA results showed significant differences between the HIP and control groups (*P* = 0.001). However, we also found that the samples from 10 pregnant women (cluster 2) showed a bacterial composition distinct from those of the rest of the study cohort (cluster 1) (pMANOVA *P* = 0.001, Fig. 1). There were no significant differences in anthropometric or biochemical variables between cluster 1 and cluster 2 (Table S1). Based on microbial analysis, it was found that the abundance of the *Bacteroidaceae* family was higher in Cluster 1 (32.1 [18.2–45.8] vs. 7.6 [0.9–14.2] %, *P* < 0.0001), whereas the proportion of the *Prevotellaceae* family was more abundant in Cluster 2 (2.6 [0–7.9] vs. 38.5 [17.0–59.9] %, *P* < 0.0001). Consistent with previous enterotype structure reports [25], Cluster 1 and Cluster 2 were classified as *Bacteroides* and *Prevotella* enterotypes. However, no significant relationship was found between enterotype and disease status (*P* = 0.103, Fisher’s exact test). Furthermore, we examined the principal components with a contribution greater than 4% and found that the first and second principal components were not only significantly correlated with enterotype (*P* < 0.0001, Mann–Whitney U test) but were also significantly correlated with disease status (*P* < 0.05, Mann–Whitney U test). These results suggested that in addition to enterotype, disease status was also an important factor affecting the gut microbial composition in our study (Table S2).

### The microbiotas of women with HIP and healthy pregnant women are different in early pregnancy

**Fig. 2:**
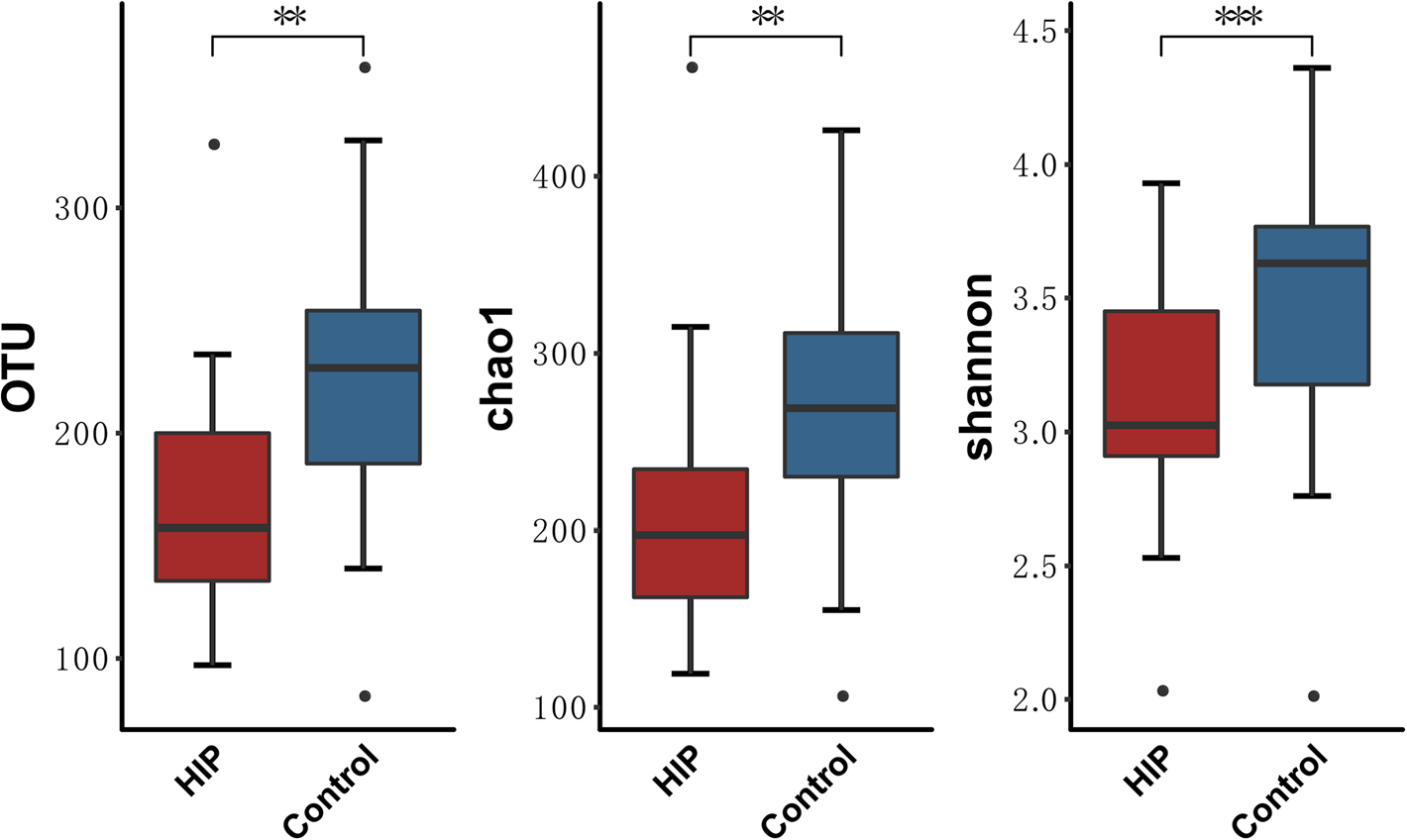
Decreased gut microbial richness and diversity in women with HIP Richness and α-diversity (Chao1 and Shannon index) of the two cohorts at the OTU level. Boxplots showing both richness and diversity values. *P < 0.05; **P < 0.01; ***P < 0.001.

We compared the gut microbial composition between 22 HIP and 28 healthy women in early pregnancy. We found that the HIP group showed significantly lower microbial richness and α-diversity than the healthy controls (*P* < 0.05 for both indexes, Mann–Whitney U test; Fig. 2, Fig. S1). PMANOVA analysis showed that there was a significant distance between the HIP and control groups (*P* < 0.001). Consistent with the PMANOVA results, CCA also showed a marked separation of women with HIP from the controls (Fig. S2).

**Fig. 3:**
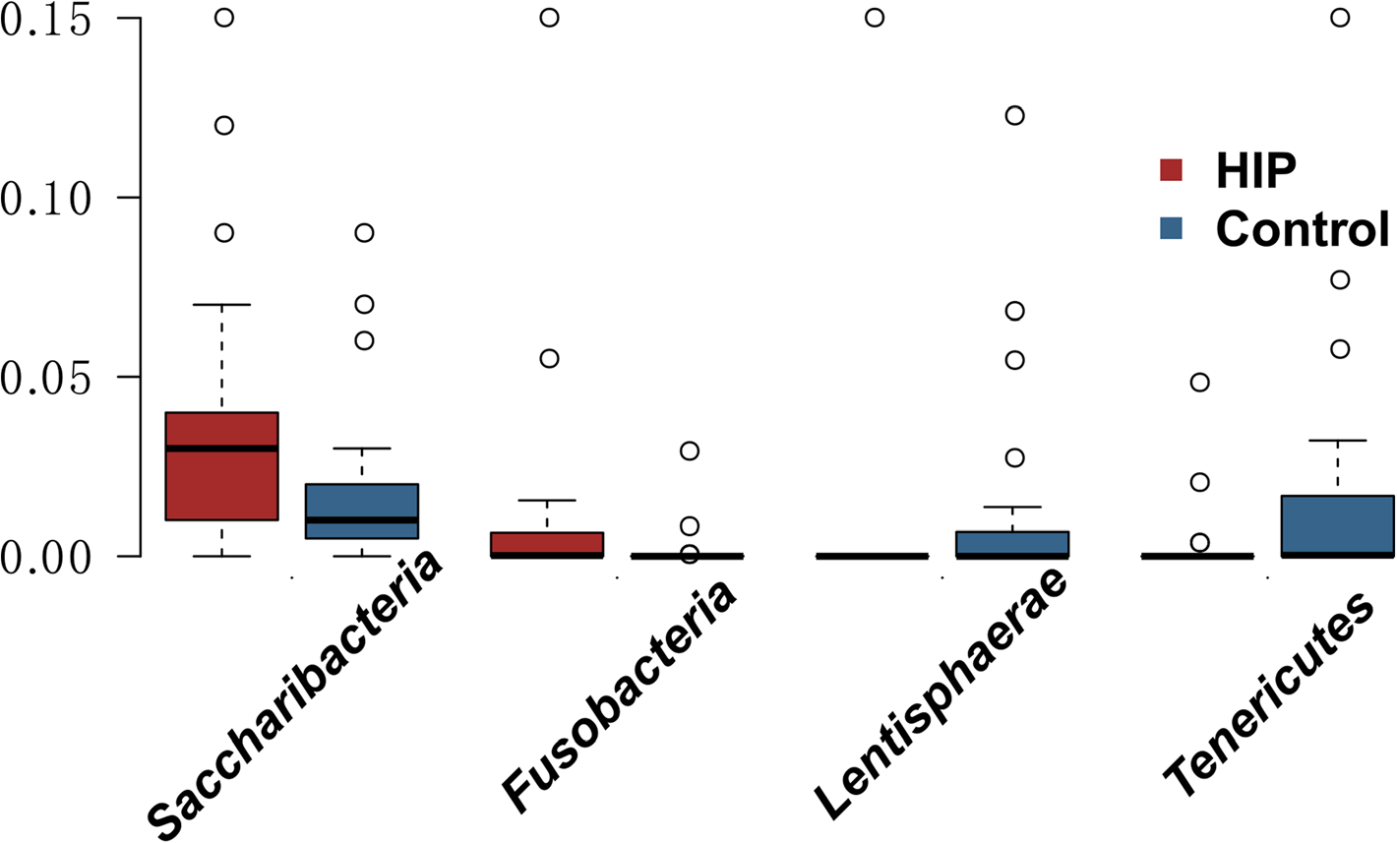
Phylum-level differences in bacteria in the faecal samples of controls and women with HIP. Boxplot shows the phyla that differed significantly between the women with HIP (red) and healthy controls (blue). The percentage of the phylum is indicated on the y axis. Boxplots showing the 25th and 75th percentiles with a line at the median

At the phylum level, *Firmicutes* and *Bacteroidetes* were the dominant phyla, accounting for ~ 91.5% of all sequences in individuals, whereas 0.04% was accounted for by unknown bacteria (Table S3). The ratio of *Firmicutes* to *Bacteroidetes* was similar in patients and controls (1.33 vs. 1.27, *P* = 0.44). Other less-prominent phyla including *Saccharibacteria* and *Fusobacteria* were enriched in the HIP group, while *Lentisphaerae* and *Tenericutes* were enriched in the healthy controls (*P* < 0.05, Fig. 3). The *Fusobacterial* class and *Fusobacteriales* order were also enriched in the HIP group.

**Fig. 4:**
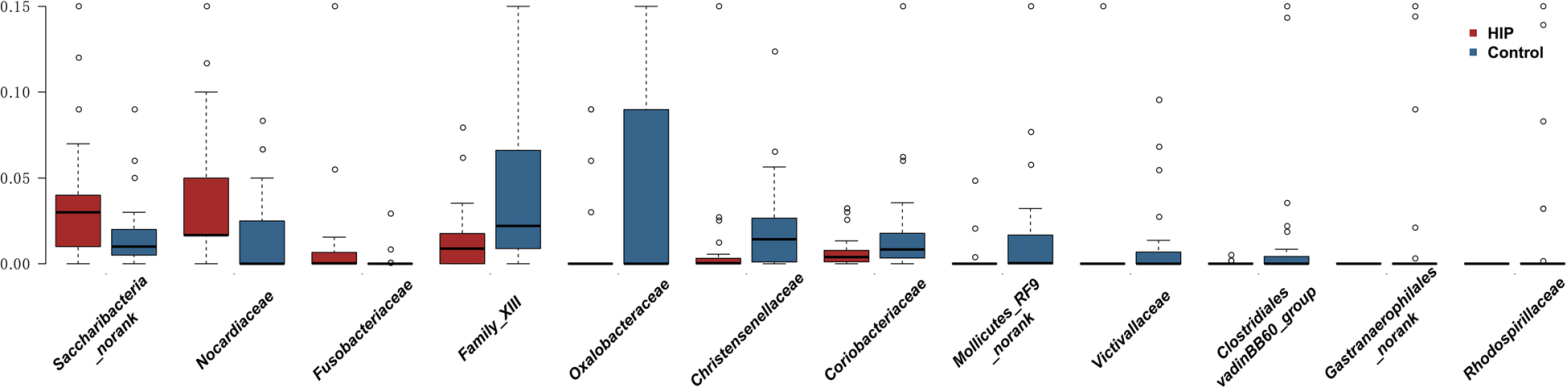
Families that differed significantly between women with HIP and healthy controls. The relative abundance of the bacterial families was significantly different between women with HIP (red) and healthy pregnant women (blue) (top 10). The percentage of microbiota is indicated on the y axis. Boxplots showing the 25th and 75th percentile with a line at the median

At the family level, women with HIP had a significantly higher proportion of *Nocardiaceae*, *Saccharibacteria_norank*, and *Fusobacteriaceae*, while the proportions of *Christensenellaceae*, *Clostridiales_vadinBB60_group*, *Mollicutes_RF9_norank*, *Oxalobacteraceae*, *Victivallaceae*, *Rhodospirillaceae*, and *Coriobacteriaceae* were higher in healthy controls (*P* < 0.05; Fig. 4). In addition, many genera showed significantly lower abundances in women with HIP than in controls, and these genera included *Ruminococcaceae_UCG-014*, *Christensenellaceae_R-7_group*, *Ruminococcaceae_UCG-003*, and *Ruminococcaceae_UCG-002*. Differences at the species level were also found between women with HIP and healthy controls and were consistent with the findings at the genus level (Table S3). These findings suggested that women with HIP had gut microbial dysbiosis.

### Correlations between the gut microbiota and clinical characteristics

**Fig. 5:**
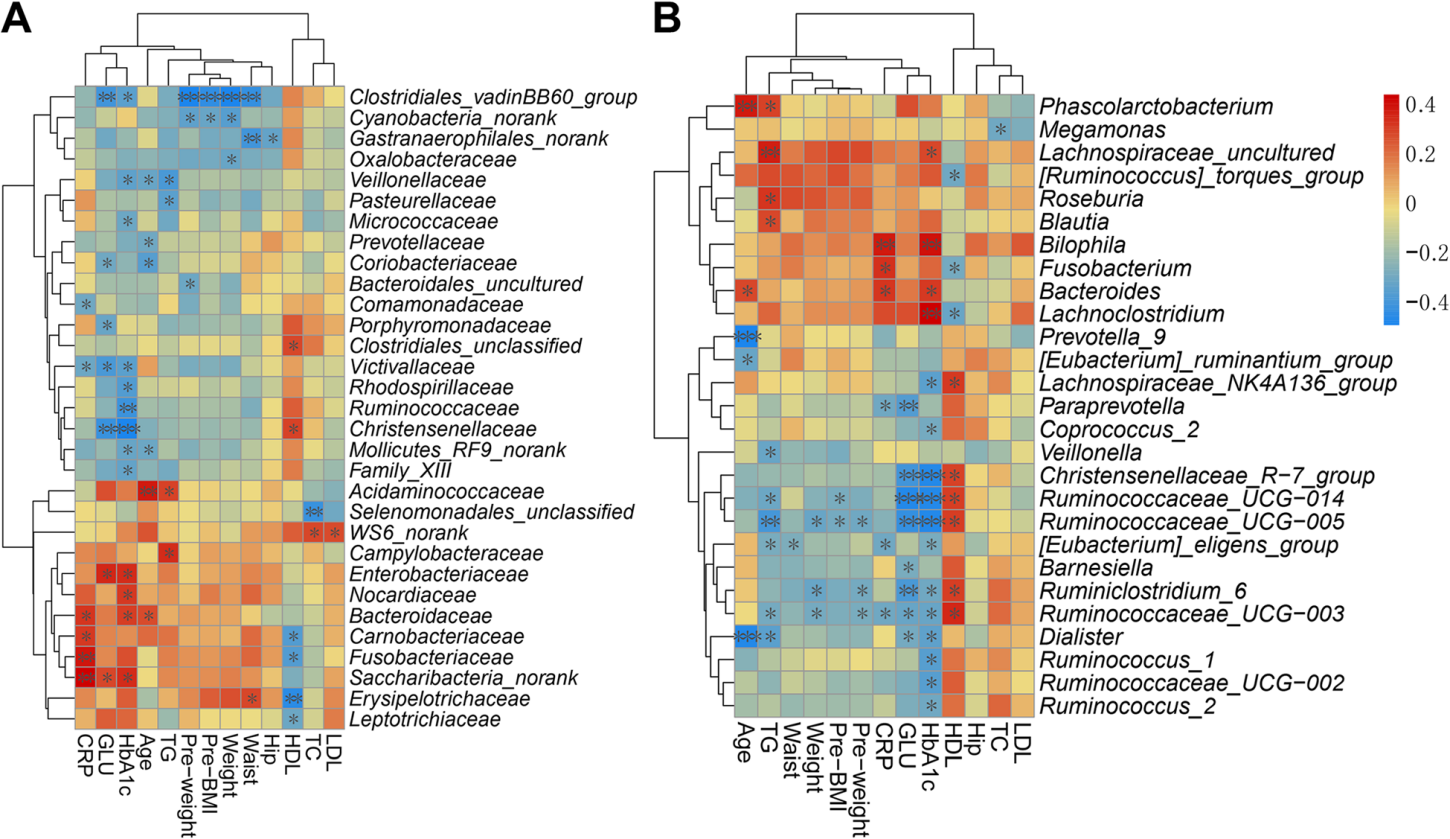
Correlation of clinical characteristics with microbial abundance Correlation analysis results of all samples at the family level (**a**) and the genus level (**b**). Spearman’s rank correlation coefficients and *P*-values for the correlations are shown. *P < 0.05; **P < 0.01; ***P < 0.001

To assess associations between clinical characteristics and the gut microbiota, Spearman’s correlation coefficient was calculated. At the family level, pre-BMI, pre-weight and waist were significantly negatively correlated with the *Clostridiales_vadinBB60_group* (Fig. 5a). pre-BMI was also negatively associated with *Ruminococcaceae_UCG-005* and *Ruminococcaceae_UCG-014*. In addition, waist was negatively correlated with the *Gastranaerophilales_norank* and positively correlated with *Erysipelotrichaceae*.

**Fig. 6:**
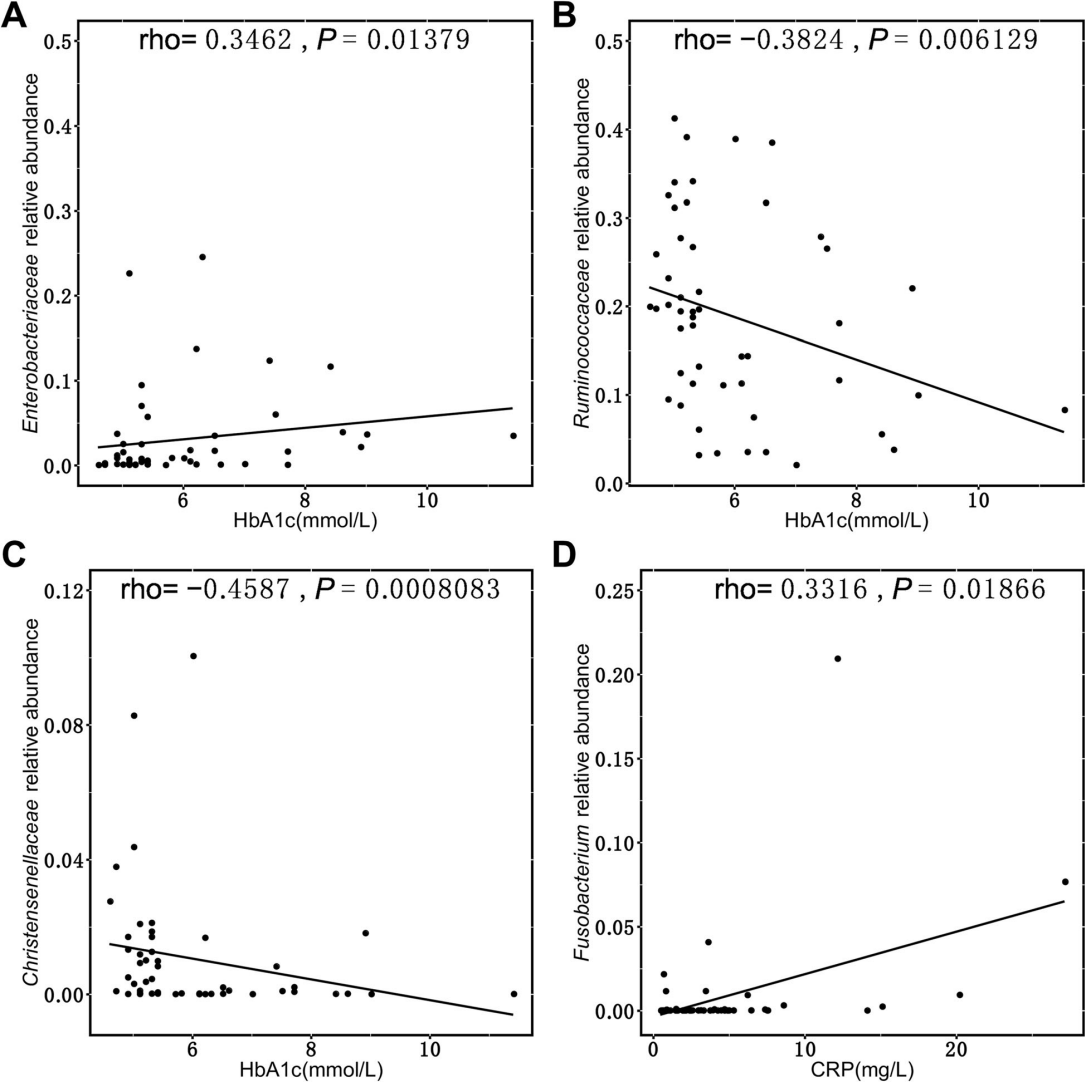
Bacterial abundance and its associations with HbA1c and CRP profile **a**. Positive correlation between HbA1c concentration and the *Enterobacteriaceae* family. **b**, **c**. Negative correlation between the *Ruminococcaceae* and *Christensenllaceae* families with HbA1c. **d**: Positive correlation between the *Fusobacterium* genus and CRP concentration

The relationship between gut microbial composition and blood glucose metabolism was also assessed. HbA1c was positively correlated with the relative abundance of *Proteobacteria*, *Fusobacteria*, and *Saccharibacteria* and negatively correlated with *Lentisphaerae* (*P* < 0.05). Furthermore, HbA1c was positively associated with the abundance of the *Bacteroidaceae* family from the *Bacteroidetes* phylum (Fig. 5a). A positive correlation between HbA1c, GLU and the *Enterobacteriaceae* family was observed (*P* < 0.05) (Fig. 6a). A higher HbA1c level was correlated with a lower abundance of multiple families, such as *Ruminococcaceae*, *Christensenellaceae*, *Victivallaceae*, *Rhodospirillaceae*, and *Micrococcaceae*, while GLU exhibited a similar trend (Fig. 5 a, Fig. 6 b, C). At the genus level, *Bacteroides*, *Bilophila* and *Lachnospiraceae_uncultured* were positively correlated with HbA1c, while several genera of the *Ruminococcaceae* family were negatively correlated with HbA1c and GLU (Fig. 5 b).

Low-grade chronic inflammation plays a key role in the pathophysiology of glucose disorders. CRP is mainly used as a marker of inflammation and has been proven to be associated with diabetes. In our study, CRP was positively correlated with the relative abundance of *Proteobacteria*, *Fusobacteria*, and *Saccharibacteria* and negatively correlated with *Lentisphaerae* (*P* < 0.05). *Fusobacteriaceae* was the main family of the *Fusobacteria* phyla. Further analysis within *Fusobacteria* indicated that the *Fusobacteriaceae* family and the *Fusobacterium* genus were also positively associated with CRP (*P* < 0.05) (Fig. 5a, Fig. 6d). We also found that the abundance of the *Bacteroidaceae* family from the *Bacteroidetes* phylum was positively associated with CRP.

## Discussion

This study is the first to our knowledge to compare the stool microbiota of patients who have hyperglycaemia (HIP) with that of controls in early pregnancy. We discovered that the participants were separated into two clusters. This finding was not explained by differences in anthropometric or biochemical variables, and the two clusters corresponded to *Bacteroides* and *Prevotella* enterotypes, respectively, in accordance with the recently-proposed theory of enterotypes [26, 27]. No significant correlation between enterotype and disease status was found in our study. However, we should fully take enterotype into account in gut microbiota research, although our current study lacks the sample size to make these conclusions. Furthermore, by examining the top six principal components, we found that the first and second principal components were also significantly correlated with disease status. We concluded that glucose metabolism disorders, in addition to enterotype, was a determining factor in explaining the gut microbial differences in our research.

We observed reduced richness and diversity in the faecal microbiota of the HIP group compared to that of the controls. Similar findings were reported for a variety of chronic diseases, including obesity [6, 7], inflammatory bowel disease [28], cancer [29] and even mental disorders [30], indicating that a decline in richness and diversity is a common characteristic in many kinds of diseases.

Most studies discovered that obesity is characterized by an increase in the *Firmicutes* phylum and a relative decrease in the *Bacteroidetes* phylum [7–9]. However, this phenomenon was quite contradictory in the context of type 2 diabetes. A study from Nadja Larsen and colleagues that included 18 male type 2 diabetes patients and 18 healthy male controls demonstrated that the relative abundance of *Firmicutes* was significantly reduced in individuals with type 2 diabetes [12]. Another study from China that included 71 type 2 diabetes patients and 74 controls also obtained similar results [24, 26]. However, the abundance of *Firmicutes* and *Bacteroidetes* and the ratio of *Firmicutes*:*Bacteroidetes* were similar between the two groups in our study. Our findings were consistent with previous research on polycystic ovary syndrome (PCOS) [31], GDM in the second trimester and GDM within 1–2 days before delivery [16, 17]. We speculate that the opposite results may be because the subjects in the latter four studies were all women who were much younger than those included in the first two studies. When comparing less-prominent phyla, we observed that *Lentisphaerae* and *Tenericutes* were enriched in healthy controls. Consistent with other studies, *Tenericutes* was more abundant in controls than in individuals with PCOS [31] or metabolic syndrome [32]. Therefore, we have reason to believe that bacteria from *Tenericutes* seem to play a protective role against several kinds of diseases.

The *Clostridiales* order, which can produce butyrate, was reduced in individuals with obesity [33], T2DM [24, 26] and gestational diabetes mellitus in the second trimester [16]. Our data supported this finding by showing that *Clostridiales* was enriched in healthy pregnant women in early pregnancy and that the *Clostridiales_vadinBB60_group* family was negatively associated with several obesity-related markers*.* Previous studies demonstrated that butyrate seemed to possess beneficial effects in terms of insulin sensitivity and energy balance [34]. The reason may partly explain why *Clostridiales* was more abundant in healthy subjects than in individuals with metabolic disease both in pregnancy and non-pregnancy conditions. The *Christensenellaceae* family, which has recently been reported in relation to leanness and healthy metabolism, was found in a significantly higher level in controls than in women with HIP and was negatively correlated with HbA1c in our study [35]. Previous studies have found that *Christensenellaceae is* negatively associated with the branch-chain amino acids (BCAAs) isoleucine and valine [34, 36]. BCAAs are positively associated with insulin resistance [37, 38]. Moreover, increased concentrations of three BCAAs (valine, leucine, isoleucine) and two aromatic amino acids (phenylalanine, tyrosine) were associated with future diabetes in the Framingham Heart Study [39]. All these results suggest that *Christensenellaceae* played a protective role against diabetes through altered amino acid metabolism.

We also observed that several genera from the *Ruminococcaceae* family were more abundant in controls than in women with HIP and were negatively correlated with HbA1c. Consistent with previous findings, *Ruminococcaceae* were depleted in women with GDM in the third trimester of pregnancy [40], in non-pregnant individuals with type 1 diabetes [41] and even in pre-diabetic subjects [36]. However, the opposite result was obtained in some other studies. Luisa F. Gomez-Arango and colleagues demonstrated that a high abundance of *Ruminococcaceae* may be related to adverse metabolic health in early pregnancy in overweight and obese women [42]. This finding was further supported by Kati Mokkala, who studied overweight and obese women in early pregnancy [43]. *Ruminococcaceae* is considered to produce short-chain fatty acids that have positive effects on metabolism [44]. The efficiency of *Ruminococcaceae* in the last two studies, which focused on overweight and obese women, may have been lower.

*Enterobacteriaceae* is a large family of Gram-negative bacteria that includes many of the familiar pathogens. A positive correlation of the *Enterobacteriaceae* family with HbA1c was observed in our study. *Enterobacteriaceae* was also found in a higher relative abundance in GDM patients in the second trimester [16] and in non-pregnant individuals with T2DM than in controls [45] and in individuals with some chronic diseases, such as colitis [46] and inflammatory bowel disease (IBD) [47]. A previous study showed that *Enterobacteriaceae* indicated a specific status of gut inflammation and colitis due to its role in inducing intestinal inflammation [46]. The level of the endotoxin-producing *Enterobacter* decreased in a morbidly obese human who lost 51.4 kg after 23 weeks of dietary intervention [48]. These results suggest that abnormalities of bacteria from the *Enterobacteriaceae* family may be associated with HIP.

The strengths of our research are that the research sample was composed of young adults with few concomitant medications and comorbidities. The main limitations of our present study are the small sample size and the fact that lifestyle and diet, which may affect both blood glucose levels and the gut microbiota, were impossible to assess in the present study.

In conclusion, our study suggests that women with HIP have dysbiosis of the gut microbiota. In particular, our results support the idea that certain members of the microbiota were associated with glucose metabolism during pregnancy. The gut microbiota may be a potential biomarker for patients with glucose metabolism disorder in early pregnancy. Future studies are needed to evaluate the diagnostic value of relevant microbial markers.

## Supplementary information

**Additional file 1: Table S1** Characteristics of Cluster 1 and Cluster 2. **Table S2** PCoA results of OTUs profiles. **Figure S1**. Richness and a-diversity (ACE and Simpson index) of the two cohorts at the OTU level. **Figure S2**. Comparison of the microbiota community composition between HIPs and healthy pregnant women

**Additional file 2 Table S3**. Differential microbial abundance between HIPs and controls

## Data Availability

The datasets used and analysed in the current study are available from the corresponding author upon reasonable request.

## Abbreviations

HIP: Hyperglycaemia in pregnancy
PGDM: Pregestational diabetes
GDM: Gestational diabetes mellitus
OGTT: Oral glucose tolerance test
T2DM: Type 2 diabetes
FLASH: Fast length adjustment of short reads
RDP: Ribosomal Database project
PMANOVA: Permutational multivariate analysis of variance
CCA: Canonical correspondence analysis
OUTs: Operational taxonomic units
PCOS: Polycystic ovary syndrome
BCAAs: Branch-chain amino acids
IBD: Inflammatory bowel disease.

## References

[1] Cosson E, Carbillon L, Valensi P. High fasting plasma glucose during early pregnancy: a review about early gestational diabetes mellitus. J Diabetes Res. 2017;2017. 10.1155/2017/8921712 PMC5664285 29181414

[2] Association AD (2018). 2. Classification and diagnosis of diabetes: standards of medical care in diabetes—2018. Diabetes Care.

[3] Lawrence JM, Contreras R, Chen W, Sacks DA. Trends in the prevalence of pre-existing diabetes and gestational diabetes mellitus among a racially/ethnically diverse population of pregnant women, 1999-2005. Diabetes Care. 2008;31(5):899–904. 10.2337/dc07-2345 18223030

[4] Dunne F, Brydon P, Smith K, Gee H. Pregnancy in women with type 2 diabetes: 12 years outcome data 1990–2002. Diabetic Med. 2003;20(9):734–8. 10.1046/j.1464-5491.2003.01017.x 12925053

[5] Bäckhed F, Ding H, Wang T, Hooper LV, Koh GY, Nagy A, et al. The gut microbiota as an environmental factor that regulates fat storage. Proc Natl Acad Sci USA. 2004;101(44):15718–23. 10.1073/pnas.0407076101 PMC524219 15505215

[6] Le Chatelier E, Nielsen T, Qin J, Prifti E, Hildebrand F, Falony G, et al. Richness of human gut microbiome correlates with metabolic markers. Nature. 2013;500(7464):541. 10.1038/nature12506 23985870

[7] Ley RE, Bäckhed F, Turnbaugh P, Lozupone CA, Knight RD, Gordon JI. Obesity alters gut microbial ecology. Proc Natl Acad Sci USA. 2005;102(31):11070–5. 10.1073/pnas.0504978102 PMC1176910 16033867

[8] Ley RE, Turnbaugh PJ, Klein S, Gordon JI. Microbial ecology: human gut microbes associated with obesity. Nature. 2006;444(7122):1022. 10.1038/4441022a 17183309

[9] Turnbaugh PJ, Ley RE, Mahowald MA, Magrini V, Mardis ER, Gordon JI. An obesity-associated gut microbiome with increased capacity for energy harvest. Nature. 2006;444(7122):1027. 10.1038/nature05414 17183312

[10] Pedersen HK, Gudmundsdottir V, Nielsen HB, Hyotylainen T, Nielsen T, Jensen BA, et al. Human gut microbes impact host serum metabolome and insulin sensitivity. Nature. 2016;535(7612):376. 10.1038/nature18646 27409811

[11] Matheus V, Monteiro L, Oliveira R, Maschio D, Collares-Buzato C. Butyrate reduces high-fat diet-induced metabolic alterations, hepatic steatosis and pancreatic beta cell and intestinal barrier dysfunctions in prediabetic mice. Exp Biol Med. 2017;242(12):1214–26. 10.1177/1535370217708188 PMC5476343 28504618

[12] Larsen N, Vogensen FK, van den Berg FW, Nielsen DS, Andreasen AS, Pedersen BK, et al. Gut microbiota in human adults with type 2 diabetes differs from non-diabetic adults. PLoS One. 2010;5(2):e9085. 10.1371/journal.pone.0009085 PMC2816710 20140211

[13] Karlsson FH, Tremaroli V, Nookaew I, Bergström G, Behre CJ, Fagerberg B, et al. Gut metagenome in European women with normal, impaired and diabetic glucose control. Nature. 2013;498(7452):99. 10.1038/nature12198 23719380

[14] Lain KY, Catalano PM. Metabolic changes in pregnancy. Clin Obstet Gynaecol. 2007;50(4):938–48. 10.1097/GRF.0b013e31815a5494 17982337

[15] Koren O, Goodrich JK, Cullender TC, Spor A, Laitinen K, Bäckhed HK, et al. Host remodeling of the gut microbiome and metabolic changes during pregnancy. Cell. 2012;150(3):470–80. 10.1016/j.cell.2012.07.008 PMC3505857 22863002

[16] Kuang Y-S, Lu J-H, Li S-H, Li J-H, Yuan M-Y, He J-R, et al. Connections between the human gut microbiome and gestational diabetes mellitus. Gigascience. 2017;6(8):1–12. 10.1093/gigascience/gix058 PMC5597849 28873967

[17] J Wang, J Zheng , W Shi, N Du, X Xu, Y Zhang, et al. Dysbiosis of maternal and neonatal microbiota associated with gestational diabetes mellitus. Gut. 2018;67(9):gutjnl-2018-315988. 10.1136/gutjnl-2018-315988 PMC6109274 29760169

[18] International Association of Diabetes and Pregnancy Study Groups Consensus Panel, Metzger BE, Gabbe SG, Persson B, Buchanan TA, Catalano PA, et al. International Association of Diabetes and Pregnancy Study Groups Recommendations on the diagnosis and classification of hyperglycemia in pregnancy. Diabetes Care. 2010;33(3):676–82. 10.2337/dc09-1848 PMC2827530 20190296

[19] Bolger AM, Lohse M, Usadel B. Trimmomatic: a flexible trimmer for Illumina sequence data. Bioinformatics. 2014;30(15):2114–20. 10.1093/bioinformatics/btu170 PMC4103590 24695404

[20] Magoč T, Salzberg SL. FLASH: fast length adjustment of short reads to improve genome assemblies. Bioinformatics. 2011;27(21):2957–63. 10.1093/bioinformatics/btr507 PMC3198573 21903629

[21] Edgar RC. Search and clustering orders of magnitude faster than BLAST. Bioinformatics. 2010;26(19):2460–1. 10.1093/bioinformatics/btq461 20709691

[22] Edgar R. UCHIME2: improved chimera prediction for amplicon sequencing. Biorxiv. 2016;074252.

[23] Wang Q, Garrity GM, Tiedje JM, Cole JR. Naive Bayesian classifier for rapid assignment of rRNA sequences into the new bacterial taxonomy. Appl Environ Microbiol. 2007;73(16):5261–7. 10.1128/AEM.00062-07 PMC1950982 17586664

[24] Quast C, Pruesse E, Yilmaz P, Gerken J, Schweer T, Yarza P, et al. The SILVA ribosomal RNA gene database project: improved data processing and web-based tools. Nucleic Acids Res. 2012;41(D1):D590–D6. 10.1093/nar/gks1219 PMC3531112 23193283

[25] Costea PI, Hildebrand F, Manimozhiyan A, Bäckhed F, Blaser MJ, Bushman FD, et al. Enterotypes in the landscape of gut microbial community composition. Nat Microbiol. 2018;3(1):8. 10.1038/s41564-017-0072-8 PMC5832044 29255284

[26] Qin J, Li Y, Cai Z, Li S, Zhu J, Zhang F, et al. A metagenome-wide association study of gut microbiota in type 2 diabetes. Nature. 2012;490(7418):55. 10.1038/nature11450 23023125

[27] Fugmann M, Breier M, Rottenkolber M, Banning F, Ferrari U, Sacco V, et al. The stool microbiota of insulin resistant women with recent gestational diabetes, a high risk group for type 2 diabetes. Sci Rep. 2015;5:13212. 10.1038/srep13212 PMC4538691 26279179

[28] Manichanh C, Rigottier-Gois L, Bonnaud E, Gloux K, Pelletier E, Frangeul L, et al. Reduced diversity of faecal microbiota in Crohn’s disease revealed by a metagenomic approach. Gut. 2006;55(2):205–11. 10.1136/gut.2005.073817 PMC1856500 16188921

[29] Chen J, Domingue JC, Sears CL. Microbiota dysbiosis in select human cancers: evidence of association and causality. Semin Immunol. 2017;32:25–34. 10.1016/j.smim.2017.08.001 PMC5660659 28822617

[30] Hsiao EY, McBride SW, Hsien S, Sharon G, Hyde ER, McCue T, et al. Microbiota modulate behavioral and physiological abnormalities associated with neurodevelopmental disorders. Cell. 2013;155(7):1451–63. 10.1016/j.cell.2013.11.024 PMC3897394 24315484

[31] Lindheim L, Bashir M, Münzker J, Trummer C, Zachhuber V, Leber B, et al. Alterations in gut microbiome composition and barrier function are associated with reproductive and metabolic defects in women with polycystic ovary syndrome (PCOS): a pilot study. PLoS One. 2017;12(1):e0168390. 10.1371/journal.pone.0168390 PMC5207627 28045919

[32] Lim MY, You HJ, Yoon HS, Kwon B, Lee JY, Lee S, et al. The effect of heritability and host genetics on the gut microbiota and metabolic syndrome. Gut. 2016;gutjnl-2015-311326. 10.1136/gutjnl-2015-311326 27053630

[33] Liu R, Hong J, Xu X, Feng Q, Zhang D, Gu Y, et al. Gut microbiome and serum metabolome alterations in obesity and after weight-loss intervention. Nat Med. 2017;23(7):859. 10.1038/nm.4358 28628112

[34] Gao Z, Yin J, Zhang J, Ward RE, Martin RJ, Lefevre M, et al. Butyrate improves insulin sensitivity and increases energy expenditure in mice. Diabetes. 2009;58(7):1509–17. 10.2337/db08-1637 PMC2699871 19366864

[35] Goodrich JK, Waters JL, Poole AC, Sutter JL, Koren O, Blekhman R, et al. Human genetics shape the gut microbiome. Cell. 2014;159(4):789–99. 10.1016/j.cell.2014.09.053 PMC4255478 25417156

[36] Org E, Blum Y, Kasela S, Mehrabian M, Kuusisto J, Kangas AJ, et al. Relationships between gut microbiota, plasma metabolites, and metabolic syndrome traits in the METSIM cohort. Genome Biol. 2017;18(1):70. 10.1186/s13059-017-1194-2 PMC5390365 28407784

[37] Stančáková A, Civelek M, Saleem NK, Soininen P, Kangas AJ, Cederberg H, et al. Hyperglycemia and a common variant of GCKR are associated with the levels of eight amino acids in 9,369 Finnish men. Diabetes. 2012; DB_111378. 10.2337/db11-1378 PMC3379649 22553379

[38] Tai E, Tan M, Stevens R, Low Y, Muehlbauer M, Goh D, et al. Insulin resistance is associated with a metabolic profile of altered protein metabolism in Chinese and Asian-Indian men. Diabetologia. 2010;53(4):757–67. 10.1007/s00125-009-1637-8 PMC3753085 20076942

[39] Wang TJ, Larson MG, Vasan RS, Cheng S, Rhee EP, McCabe E, et al. Metabolite profiles and the risk of developing diabetes. Nat Med. 2011;17(4):448. 10.1038/nm.2307 PMC3126616 21423183

[40] Crusell MKW, Hansen TH, Nielsen T, Allin KH, Rühlemann MC, Damm P, et al. Gestational diabetes is associated with change in the gut microbiota composition in third trimester of pregnancy and postpartum. Microbiome. 2018;6(1):89. 10.1186/s40168-018-0472-x PMC5952429 29764499

[41] Huang Y, Li S-C, Hu J, Ruan H-B, Guo H-M, Zhang H-H, et al. Gut microbiota profiling in Han Chinese with type 1 diabetes. Diabetes Res Clin Pract. 2018;141:256–63. 10.1016/j.diabres.2018.04.032 29733871

[42] Gomez-Arango LF, Barrett HL, McIntyre HD, Callaway LK, Morrison M, Nitert MD, et al. Connections between the gut microbiome and metabolic hormones in early pregnancy in overweight and obese women. Diabetes. 2016:db160278. 10.2337/db16-0278 27217482

[43] Mokkala K, Houttu N, Vahlberg T, Munukka E, Rönnemaa T, Laitinen K. Gut microbiota aberrations precede diagnosis of gestational diabetes mellitus. Acta Diabetol. 2017;54(12):1147–9. 10.1007/s00592-017-1056-0 28980079

[44] Bearson SMD, Allen HK, Bearson BL, Looft T, Brunelle BW, Kich JD, et al. Stanton TB: profiling the gastrointestinal microbiota in response to Salmonella : low versus high Salmonella shedding in the natural porcine host. Infect Genet Evol. 2013;16:330–4. 10.1016/j.meegid.2013.03.022 23535116

[45] Salamon D, Sroka-Oleksiak A, Kapusta P, Szopa M, Mrozińska S, Ludwig-Słomczyńska AH, et al. Characteristics of gut microbiota in adult patients with type 1 and type 2 diabetes based on next-generation sequencing of the 16S rRNA gene fragment. Pol Arch Med Wewn. 2018;128(6):336–43. 10.20452/pamw.4246 29657308

[46] Garrett WS, Gallini CA, Yatsunenko T, Michaud M, DuBois A, Delaney ML, et al. Enterobacteriaceae act in concert with the gut microbiota to induce spontaneous and maternally transmitted colitis. Cell Host Microbe. 2010;8(3):292–300. 10.1016/j.chom.2010.08.004 PMC2952357 20833380

[47] Morgan XC, Tickle TL, Sokol H, Gevers D, Devaney KL, Ward DV, et al. Dysfunction of the intestinal microbiome in inflammatory bowel disease and treatment. Genome Biol. 2012;13(9):R79. 10.1186/gb-2012-13-9-r79 PMC3506950 23013615

[48] Fei N, Zhao L. An opportunistic pathogen isolated from the gut of an obese human causes obesity in germfree mice. ISME J. 2013;7(4):880. 10.1038/ismej.2012.153 PMC3603399 23235292

