# Supplementary material for: Gut microbiota in early pregnancy among women with Hyperglycaemia vs. Normal blood glucose
Source: BMC Pregnancy Childbirth. 2020 May 11;20:284. doi: 10.1186/s12884-020-02961-5 (PMC7216510; doi:10.1186/s12884-020-02961-5)
Supplement: Supplementary file 1 — Additional file 1: Table S1 Characteristics of Cluster 1 and Cluster 2. Table S2 PCoA results of OTUs profiles. Figure S1. Richness and a-diversity (ACE and Simpson index) of the two cohorts at the OTU level. Figure S2. Comparison of the microbiota community composition between HIPs and healthy pregnant women [file 12884_2020_2961_MOESM1_ESM.docx]

**TableS1 Characteristics of Cluster 1 and Cluster 2**

| **Term** | **Cluster 1 (n=40)** | **Cluster 2 (n=10)** | **PValue** |
| --- | --- | --- | --- |
| Diabetes(YES/NO) | 13/27(32.5%/67.5%) | 3/7(30%/70%) | 1 # |
| Obesity(YES/NO) | 2/38(5%/95%) | 1/9(10%/90%) | 0.496 # |
| Age | 29.6(25.32-33.88) | 27.2(23.81-30.59) | 0.11 |
| BMI | 23.15(18.6-27.7) | 23.93(18.99-28.87) | 0.472 |
| Pre-weight | 59.63(46.86-72.39) | 61(47.39-74.61) | 0.527 |
| Waist | 88.15(75.77-100.53) | 91.9(80.38-103.42) | 0.244 |
| Hip | 95.15(87.54-102.76) | 99.95(91.72-108.18) | 0.0847 |
| GLU | 5.08(3.76-6.4) | 5.85(3.68-8.03) | 0.56 |
| TC | 4.42(3.72-5.12) | 4.38(3.3-5.45) | 0.513 |
| TG | 1.59(0.95-2.23) | 2.69(0.56-4.82) | 0.505 |
| HDL | 1.63(1.23-2.02) | 1.61(1.01-2.22) | 0.654 |
| LDL | 2.56(1.91-3.21) | 2.27(1.67-2.87) | 0.0918 |
| HBA1C | 6.04(4.6-7.47) | 5.86(4.6-7.12) | 0.644 |
| CRP | 4.82(-0.62-10.25) | 3.42(-0.61-7.46) | 0.244 |

Data are represented as median (interquartile range), unless otherwise stated. Mann-Whitney U tests were used except for #Fisher Exact test. GLU, fasting blood glucose; TC, serum total cholesterol; TG, triglyceride; HbA1c, glycated hemoglobin.

**Table S2 PCoA results of OTUs profiles**

| PCoA | Enterotypes | HIP | BMI | Age |
| --- | --- | --- | --- | --- |
| 1 | **1.95E-10** | **0.0366** | 0.687 | 0.072 |
| 2 | **0.025** | **0.0406** | 0.923 | 0.739 |
| 3 | 0.274 | 0.077 | 0.427 | 0.665 |
| 4 | 0.711 | 0.794 | 0.800 | 0.317 |
| 5 | 0.607 | 0.239 | 0.473 | **0.022** |
| 6 | 0.784 | 0.141 | 0.077 | 0.781 |

The top six principle components (PCs) were tested for correlations with some known factors, such as enterotypes, HIP, BMI and age and significant correlation show in bold form.

**Figure S1. Richness and a-diversity (ACE and Simpson index) of the two cohorts at the OTU level**

Comparison of gut microbiota diversity within HIP (red) and healthy (blue) pregnant women estimated by ACE, Coverage and Simpson index. Boxplots showing the 25^th^ and 75^th^ percentile with a line at median. Boxplots showing both the richness or diversity values. *P < 0.05; **P < 0.01; ***P < 0.001.

**Figure S2. Comparison of the microbiota community composition between HIPs and healthy pregnant women**

A CCA plot shows clustering of bacterial populations at the OTU level according to diseases categories. The percentage of variation is shown. It suggests that HIP significantly affects gut microbiota composition (P= 0.009).
